# The Effect of Exenatide on Platelets Ratio Index and Fibrosis-4 Index in Obese Patients With Diabetes Mellitus

**DOI:** 10.1155/ije/6332117

**Published:** 2025-04-28

**Authors:** Gamze Ergun Sezer, Meral Mert

**Affiliations:** ^1^Department of Nephrology, Bakirkoy Dr. Sadi Konuk Education and Research Hospital, University of Health Sciences, Zuhuratbaba, Tevfik Saglam Avenue. No: 11, Bakirkoy, Istanbul, Turkey; ^2^Department of Endocrinology, Bakirkoy Dr. Sadi Konuk Education and Research Hospital, University of Health Sciences, Zuhuratbaba, Tevfik Saglam Avenue. No: 11, Bakirkoy, Istanbul, Turkey

**Keywords:** APRI, exenatide, FIB-4, obesity, type 2 diabetes mellitus

## Abstract

Many studies have shown a close relationship between type 2 diabetes mellitus and obesity. Glucagon-like peptide-1 (GLP-1) agonists are particularly preferred as antidiabetic medications for obese patients with type 2 diabetes because they not only help with glycemic control but also promote weight loss by slowing gastric emptying. Fatty liver disease, a significant complication of obesity, can progress to hepatic fibrosis and cirrhosis in later stages. Platelets ratio index (APRI), fibrosis-4 index (FIB-4), indices are two of the most studied indirect markers of hepatic fibrosis. Our study aimed to investigate the effect of exenatide, a GLP-1 agonist, on the APRI and FIB-4 indices in obese patients with type 2 diabetes mellitus. We included obese patients with type 2 diabetes treated with exenatide at the endocrinology and metabolism outpatient clinics of Bakırköy Dr. Sadi Konuk Training and Research Hospital between January 2015 and May 2018. We calculated the APRI and FIB-4 indexes retrospectively using data on aspartate aminotransferase, alanine aminotransferase, platelet counts, and ages. The study included 170 patients, with an average age of 48.27 ± 11 years. We compared the APRI and FIB-4 indices at the third and sixth months after the onset of exenatide and before the treatment. While there was no significant change in the FIB-4 index with treatment, the APRI index showed a significant decrease. In conclusion, our study observed a significant decrease in the APRI index with exenatide treatment, while the FIB-4 index remained unchanged. More research is needed on liver fibrosis indices in the obese population.

## 1. Introduction

Diabetes mellitus (DM) is a chronic metabolic disease that requires continuous medical care and is caused by disorders in carbohydrate, protein and fat metabolism due to complete or partial insufficiency in insulin secretion and/or effect [1]. Important studies have determined that type 2 DM is more common in obese individuals and that the risk of developing the disease is parallel to the increase in fat mass measured as body mass index (BMI) [2]. Exenatide is a glucagon-like peptide-1 (GLP-1) agonist that is particularly preferred for obese patients with type 2 diabetes [3]. This antidiabetic medication regulates blood sugar through several mechanisms, including: stimulating insulin secretion in response to glucose, suppressing glucagon secretion in a glucose-dependent manner, slowing gastric emptying, improving the function of beta cells, increasing beta cell mass, and promoting islet cell neogenesis. Additionally, exenatide is associated with reduced food intake and decreased body weight [4]. Nonalcoholic fatty liver disease (NAFLD) is defined as a condition in which 5% or more of the liver is fatty, as determined by histology or imaging, without any other causes, such as heavy alcohol consumption or hereditary disorders. It is a common condition that can progress to liver cirrhosis in its advanced stages [5, 6]. Obesity is the most significant risk factor for developing this disease. Research has shown that hepatic steatosis significantly decreases with weight loss [7]. Various studies have identified markers for predicting liver fibrosis, utilizing age, transaminases, and platelet counts. Among these, the two most studied markers are the aspartate aminotransferase (AST) to platelet ratio index (APRI), which is calculated using AST and platelet count, and the fibrosis-4 index (FIB-4), which incorporates AST, alanine aminotransferase (ALT), platelet count, and age [8, 9].

Our study aimed to investigate the effects of exenatide, a GLP-1 agonist, on APRI and FIB-4 indexes, indirect indicators of liver fibrosis, in an obese patient population with type 2 diabetes.

## 2. Methods

The study was conducted retrospectively after receiving approval from the Clinical Research Ethics Committee (Decision No: 2018/13, Date: July 23, 2018). The study involved obese type 2 diabetic patients who were treated with exenatide and visited the Endocrinology and Metabolism outpatient clinics of Bakirkoy Dr. Sadi Konuk Education and Research Hospital from January 1, 2015, to May 1, 2018. Patients included in the study were required to be over 18 years old, have a BMI greater than 35, and regularly use exenatide during their follow-ups. Those excluded from the study comprised pregnant women, patients with acute or chronic liver disease, individuals with type 1 DM, patients who did not attend regular follow-ups, and those under 18 years of age. In total, 170 patients were included in the study. The data collected included gender, age, any additional diseases, AST, ALT, platelet count, height, and weight of the participants. The APRI and FIB-4 indexes were assessed before treatment and at the third and sixth months after initiating treatment. These indexes were recorded in the system, calculated, and compared. The formulas for these calculations are as follows: FIB-4: age ^∗^ AST/platelet count ^∗^ √ ALT, APRI index: AST/platelet count [8, 9].

NCSS 2020 Statistical Software, Utah, USA programme was used for statistical analysis in evaluating the data obtained from the study. During the evaluation of the data obtained from the study, Kolmogorov–Smirnov test was used regarding the comparisons of descriptive statistical methods (mean, standard deviation, median, frequency, IQR) as well as conformity of the data to a normal distribution. Repeated Measures test was used in evaluations of normally distributed BMI according to follow-ups, and Bonferroni test was used in post hoc evaluations. For APRI and FIB-4 parameters that did not show normal distribution, Friedman's two-way analysis of Variance by ranks test was used for evaluations according to follow-up, and Bonferroni correction for multiple tests was used for post hoc evaluation. Results were given in 95% confidence interval and significance was accepted at *p* < 0.05 level.

## 3. Results

The study included 144 female patients (84.71%) and 26 male patients (15.29%) with obese type 2 DM. The mean age of these patients was 48.27 ± 11. We defined patients with BMI < 40 as obese and those with BMI > 40 as morbidly obese. BMI could not be calculated for 46 patients who did not regularly monitor their weight. The mean BMI was 45.75 ± 7.84. Of the 105 female patients, 81 were morbidly obese (77.14%), 24 were obese (22.85%), and of the 19 male patients, 14 were morbidly obese (73.68%), and 5 were obese (26.31%). Of these 170 patients, 80 had no additional disease (47.06%), and 90 had additional diseases (52.94%).

**Figure 1:**
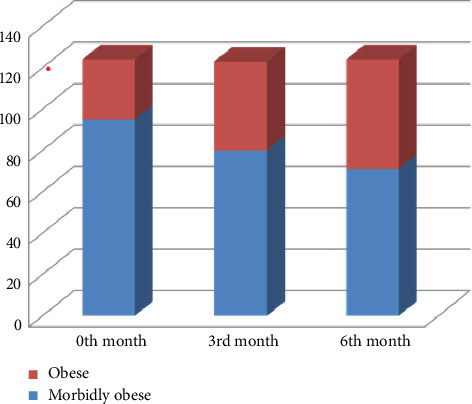
Comparison of body mass indexes at 0, 3 and 6 months.

Afterward, with exenatide treatment, the BMIs of the patients at 0, 3, and 6 months of treatment were compared. As seen in Figure 1, the morbid obesity rate of the patients decreased with the treatment.

**Figure 2:**
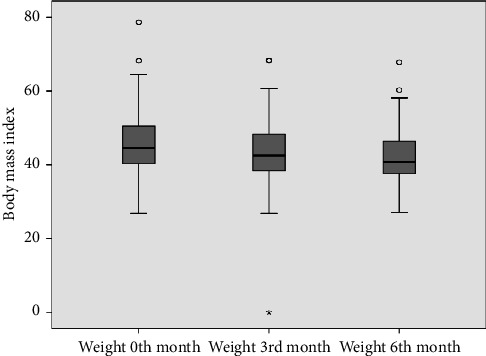
Distribution of BMI levels according to follow-ups.

As seen in Table 1 and Figure 2, there was a significant decrease in weight with exenatide treatment (*p*=0.001). Inspite of this no significant difference was found when the FIB-4 indexes of the patients were compared before treatment and at the third and sixth months after treatment (*p*: 0.455) as shown in Table 2. But significant decrease was observed between the APRI indexes before, third and sixth months after treatment (*p* : 0.004) (Table 3).

BMI levels show statistically significant differences according to follow-ups (F: 146.217; *p*=0.001); when significances were examined, BMI level at 0th month showed an average difference of 2.31 units at third month, which was found to be statistically significant (*p*=0.001). An average decrease of 3.35 units at sixth month compared to 0th month was also found to be statistically significant (*p*=0.001). A decrease of 1.04 units at sixth month compared to third month was also found to be significant (*p*=0.027).

FIB-4 levels do not show statistically significant differences according to follow-ups (Chi-square: 1574; *p*=0.453).

**Figure 3:**
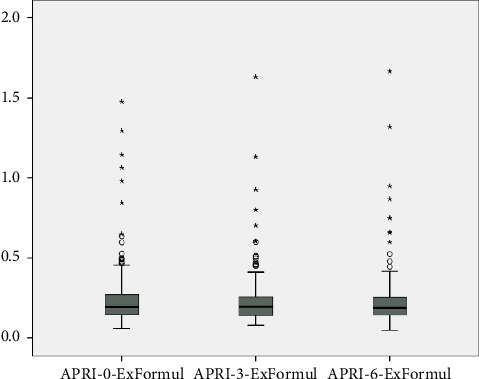
Distribution of APRI levels according to follow-ups.

APRI levels show statistically significant differences according to follow-ups (Chi-square: 10.937; *p*=0.004); when significances were examined, APRI level at 0th month showed an average difference of 0.015 units at third month, which was found to be statistically significant (*p*=0.003). No significant difference was found between 0th month and sixth month (*p* > 0.05). The decrease of 0.01 units at sixth month compared to third month was also found to be significant (*p*=0.041) (Table 3, Figure 3).

## 4. Discussion

Our results indicated a significant decrease in APRI index following exenatide treatment, but there was no change in FIB-4 index.

Obesity is a complex condition that impacts more than just an individual's weight. While BMI is a commonly used tool for diagnosing obesity, it does not fully represent the range of health issues linked to excess weight. The complications associated with obesity are comparable to those seen in other chronic diseases, leading to increased rates of morbidity and mortality [10, 11].

Numerous studies have demonstrated a positive correlation between obesity and NAFLD. Also, research consistently shows that obese individuals exhibit more severe hepatic inflammation and fibrosis compared to nonobese individuals. A recent study found that obese NAFLD patients had higher NAFLD Activity Scores (NAS), increased rates of hepatocellular ballooning—an indicator of hepatic inflammation—and more advanced fibrosis stages than their nonobese counterparts [12]. This correlation has been confirmed by a meta-analysis of eight observational studies, which indicated that overweight or obese individuals with NAFLD had more severe histological lesions, including higher NAS and fibrosis, compared to lean NAFLD patients [13]. The risk of nonalcoholic steatohepatitis (NASH) was also lower in lean individuals, with an odds ratio of 0.6 compared to overweight or obese individuals [13]. An early autopsy study from the late 1980s reported that NASH was found in 18.5% of severely obese patients, compared to only 2.7% of lean individuals. Additionally, severe fibrosis was observed in 13.8% of severely obese patients versus 6.6% of lean individuals [14]. In an Italian study, the rates of NASH were higher in overweight or obese individuals (40.9%) than in those of normal weight (17.5%) who underwent liver biopsy. Significant fibrosis (fibrosis stage [F] ≥ 2) was also more prevalent in the overweight/obese group (42.0%) than in the normal weight group (17.5%) [15]. In a similar study conducted in the U.S., 80% of obese individuals undergoing liver biopsy were found to have NASH [16]. Several longitudinal studies further support the link between obesity and the progression of significant fibrosis. One 5 year prospective study with paired liver biopsies showed that obesity was more common in patients who experienced fibrosis progression (86%) compared to those who did not (27%). Notably, the only factor independently associated with fibrosis progression in this study was BMI [17]. Another 13 year prospective study with paired liver biopsies demonstrated that weight gain exceeding 5 kg, higher insulin resistance, and more pronounced hepatic steatosis were linked to fibrosis progression [18]. This observation validates the parallel trends of hepatic steatosis and fibrosis in NAFLD, at least until the disease progresses to NASH-related cirrhosis. The relationship between obesity, NASH, and hepatic fibrosis has clinical implications; weight and BMI have been incorporated into some algorithms for the noninvasive prediction of NASH and advanced fibrosis [19–23].

Various studies have shown that exenatide has positive effects on weight loss in obese type 2 diabetic patients [24–26]. In our study, the rate of morbid obesity gradually decreased with exenatide treatment, which supports other studies. A dynamic positron emission tomography study demonstrated that exenatide reduces both hepatic and adipose tissue insulin resistance, which are key factors in the development of NAFLD [27]. In a prospective 7-month uncontrolled study involving paired liver biopsies, exenatide doses of 5–10 μg twice daily yielded variable outcomes: the NAS score improved in three out of eight patients, while fibrosis improved in four, remained stable in three, and worsened in one patient [28]. In a 6 month randomized controlled trial (RCT), exenatide was found to be more effective than other standard anti-diabetic treatments in reducing weight and hepatic fat [29]. It also showed superior results to metformin in lowering weight and liver function tests after 3 months of treatment [30]. In a 1-year RCT, the combination of exenatide and pioglitazone was more effective than pioglitazone alone in reducing ALT levels and hepatic fat, as evaluated by magnetic resonance spectroscopy (MRS), when used alongside metformin in obese patients with type 2 DM (T2DM). Notably, the weight gain observed in the pioglitazone group was less pronounced in the group receiving the combination of exenatide and pioglitazone [31]. Additionally, another 4-month RCT showed that the combination of exenatide and insulin glargine effectively decreased liver function tests and reversed hepatic steatosis (assessed through ultrasonography) more efficiently than intensive insulin therapy (which included insulin glargine and insulin aspart) in obese patients with T2DM and NAFLD [32].

Researchers have tried to develop indices to assess liver fibrosis in NAFLD patients to avoid liver biopsy because NAFLD is the most common cause of liver disease in the world. In a study published in 2021, FIB-4, APRI, and AST/ALT ratio were compared with FibroScan in the evaluation of liver fibrosis in patients with NAFLD. APRI seems to be the most suitable alternative to FibroScan for detecting significant fibrosis in patients with NAFLD. FIB-4 is the second most effective option, indicating that if FibroScan is unavailable, both APRI and FIB-4 are the best indices for assessing liver fibrosis in these patients [33]. In another study, the FIB-4 index was found to be more useful than the APRI index in detecting advanced liver fibrosis [34]. In a study published in 2008, APRI and FIB-4 index were compared in predicting hepatic fibrosis staged by liver biopsy in 81 HIV and HCV co-infected patients and FIB-4 was found to be superior [35]. Similar to our results, a decrease in the APRI index was observed with treatment, in a study including 100 diabetic patients published in 2023 [36]. Despite the conflicting results in various studies, in our study, there was a decrease in the APRI index but not in the FIB-4 index with exenatide treatment.

This study supports existing research regarding weight loss in obese and morbidly obese patients using exenatide. Although the APRI index includes fewer variables, it was found to be more sensitive than the FIB-4 index. However, our study has several limitations. We did not perform liver biopsies to diagnose NAFLD; instead, we only examined noninvasive tests, and the study was retrospective. Therefore, more comprehensive prospective studies are necessary on this topic.

## Figures and Tables

**Table 1:** Comparison of patients' weight over time.

	BMI (*n* = 124) Mean ± SD	Repeated measures test *p*	Post hoc test
Weight 0th month	45.75 ± 7.83	*F*: 146,217 *p* : 0.001^∗∗^	0th > 3rd *p*=0.001^∗∗^
Weight 3rd month	43.43 ± 8.25		0th > 6th *p*=0.001^∗∗^
Weight 6th month	42.39 ± 6.99		3rd > 6rd *p*=0.027^∗^

*Note:* Repeated measures test and post hoc Bonferroni test.

^∗^ *p* < 0.05.

^∗∗^ *p* < 0.01.

**Table 2:** Comparison of FIB-4 indexes.

	FIB4 Median (IQR)	Friedman test *p*	Post hoc test
FIB4-0th month	0.62 (0.49–0.89)	0.453	NS
FIB4-3rd month	0.64 (0.49–0.87)		
FIB4-6th month	0.65 (0.48–0.85)		

*Note:* IQR: 25%–75% percentiles; FIB-4: Fibrosis-4 index. Friedman's two-way analysis of variance by ranks.

**Table 3:** Comparison of APRI indexes.

	APRI Median (IQR)	Friedman test *p*	Post hoc test
APRI-0th month	0.19 (0.15–0.27)	0.004^∗∗^	0th > 3rd *p*=0.003^∗∗^
APRI-3rd month	0.20 (0.14–0.26)		0th > 6rd *p*=0.221
APRI-6th month	0.18 (0.14–0.25)		3rd > 6rd *p*=0.041^∗^

*Note:* IQR: 25%–75% percentiles; APRI: platelets ratio index. Friedman's two-way analysis of variance by ranks and Bonferroni correction for multiple tests.

^∗^ *p* < 0.05.

^∗∗^ *p* < 0.01.

## Data Availability

The datasets generated and/or analysed during the current study are available from the corresponding author on reasonable request.
